# Factors affecting healthcare access for dysmenorrhoea: a scoping review protocol

**DOI:** 10.1136/bmjopen-2025-100273

**Published:** 2025-08-06

**Authors:** Fódhla Ní Chéileachair, Line Caes, Sophie Belfield, Marion Bartl, Hannah Durand

**Affiliations:** 1 Division of Psychology, Faculty of Natural Sciences, University of Stirling, Stirling, UK; 2 Department of Psychology, Cornell University, Ithaca, New York, USA; 3 Insight Research Ireland Centre for Data Analytics, University College Dublin, Dublin, Ireland

**Keywords:** Dysmenorrhea, Health Services Accessibility, Health

## Abstract

**Abstract:**

**Introduction:**

Dysmenorrhoea (period pain) is a global public health issue affecting up to 91% of the 1.8 billion individuals who menstruate. While research has emphasised the improvement of menstrual health in low-middle-income countries, undertreated dysmenorrhoea remains an issue in high-income countries (HICs), where individuals often assume their pain experiences are normal. Studies report that individuals with dysmenorrhoea delay seeking medical care, avoid it entirely or are subjected to diagnostic and treatment delays. Difficulties accessing care are troubling, as individuals may suffer without access to evidence-based techniques, as well as the potential for underlying pathologies (eg, endometriosis, pelvic inflammatory disease) to go undiagnosed.

Many HICs have launched strategies for women’s health to address gaps in care access and knowledge around menstruation. Guided by Levesque and colleagues' (2013) Conceptual Framework of Access to Healthcare, this review will contribute to these strategies by providing an overview of factors affecting healthcare access for dysmenorrhoea in HICs from the point of perceiving a healthcare need to engaging with care, as well as factors affecting perceived quality of care.

**Methods and analysis:**

This scoping review will follow the Joanna Briggs Institute’s (JBI) guidance for scoping reviews and will be conducted with the Preferred Reporting Items for Systematic Reviews and Meta-Analyses checklist extension for Scoping Reviews. Guided by Levesque and colleagues’ (2013) Conceptual Framework of Access to Healthcare, searching will seek to locate both peer-reviewed studies across PubMed, CINAHL, PsycINFO and Web of Science databases, as well as using web scraping to locate relevant grey literature. Results will be synthesised and mapped to construct a pathway to care, highlighting factors affecting the healthcare access for dysmenorrhoea, as well as factors related to the quality of healthcare interactions.

**Ethics and dissemination:**

This review does not require ethical approval, as only existing data will be analysed. Results will be shared using peer-reviewed publications and conference presentations. Datasets emerging from the study will be made available on Open Science Framework.

**Registration:**

This review was initially registered on Open Science Framework (https://osf.io/2dsrc/) in February 2024, with an updated protocol registered in February 2025.

**STRENGTHS AND LIMITATIONS OF THIS STUDY**

- This review will thoroughly map the pathway to healthcare access for period pain using a comprehensive conceptual framework.
- Analysis of peer-reviewed evidence will be supplemented by extensive mapping of available grey literature sources using Python-based web scraping techniques.
- This study will involve stakeholder and expert consultation.
- This scoping review will not include a formal assessment of study quality, and, as such, studies of varying quality will be included.
- This review is limited to data available in English only.

## Introduction

 Dysmenorrhoea or ‘period pain’ is moderate to severe pelvic and/or abdominal pain during menstruation.^1^ Estimated to affect between 16 and 91% of girls, women and individuals who menstruate (*hereafter:* individuals who menstruate)^2^ dysmenorrhea can have debilitating effects on mental health,^3^ quality of life^4^ and can substantially limit participation in daily activities.^5 6^ Menstrual pain is often accompanied by other adverse symptoms, like back pain and vomiting,^7^ and may be ‘secondary dysmenorrhea,’ where pain is symptomatic of an underlying gynaecological condition, such as endometriosis, fibroids or adenomyosis.^8^,^10^

Dysmenorrhoea is a public health issue^11^ and presents a challenge to achieving menstrual health, defined by Hennegan and colleagues^12^ as ‘a state of complete physical, mental and social well-being and not merely the absence of disease or infirmity.’ Many studies have focused on improving menstrual health in low-middle-income countries, with an emphasis on ‘Menstrual Health and Hygiene’.^13^ Achieving menstrual health in high-income countries (HICs), however, remains contentious particularly around issues of dysmenorrhoea and pain management. As well as negatively affecting well-being, dysmenorrhoea is a leading cause of workplace and educational absenteeism and ‘presenteeism,’ where an individual is present but limited in their capacity to engage.^7 14 15^ Estimates from the Netherlands indicate an average annual loss of 1.3 and 8.9 days of workplace productivity due to dysmenorrhoea absenteeism and presenteeism, respectively.^16^ Recently, a report from the phs Group^14^ in the UK suggests that up to 11 academic weeks can be lost due to menstrual concerns between the ages of 13–18 years, where dysmenorrhoea was associated with 82% of absences surveyed.

Many HICs, such as Ireland,^17^ the UK,^18^ Austria^19^ and Australia,^20^ have recently produced Women’s Health Strategies, which seek to address some of these gaps in achieving menstrual health. A persistent gap is that individuals who menstruate substantially lack menstrual health literacy, operationalised as the degree to which an individual can understand, obtain and process health information pertinent to menstrual health.^21^ Individuals report learning about pain management for dysmenorrhoea through trial and error^22^ and may rely on painkillers that do not provide adequate relief.^23 24^ This issue is compounded by the fact that most individuals reportedly delay seeking healthcare or do not seek healthcare at all to manage dysmenorrhoea.^45 25^,^27^

In many HICs, seeking medical care is the only pathway forward to accessing stronger pain relief or contraception for menstrual concerns^28^,^30^ or for investigating potential underlying causes of symptoms.^31^ Among those who do seek medical care, however, pain invalidation has been reported,^32^,^34^ as well as individuals facing diagnostic delays spanning years before receiving a definitive diagnosis and appropriate care.^31 35^ Overall, while healthcare should be available to individuals with either primary or secondary dysmenorrhoea, experiences of seeking care and its consequences are poor across HICs, with a wide variety of factors identified as possible contributors to negative care experiences.^25 36 37^

### Healthcare access for dysmenorrhoea

While not all individuals with dysmenorrhoea will require healthcare advice or intervention, consulting healthcare practitioners (HCPs) may provide some individuals with evidence-based techniques they might not otherwise find, due to low menstrual health literacy rates.^21^ Absences or delays in seeking and receiving care from healthcare practitioners broadly represent issues of *‘healthcare access,’* which is defined by Levesque and colleagues^38^ as the opportunity to reach appropriate healthcare and receive services to fulfil a perceived healthcare need. The framework by Levesque and colleagues operationalises healthcare access as an overarching pathway towards fulfilling a perceived healthcare need, where both health system (supply-side) factors and individual (demand-side) factors engage and interact to either promote or impede successful healthcare access.

**Figure 1:**
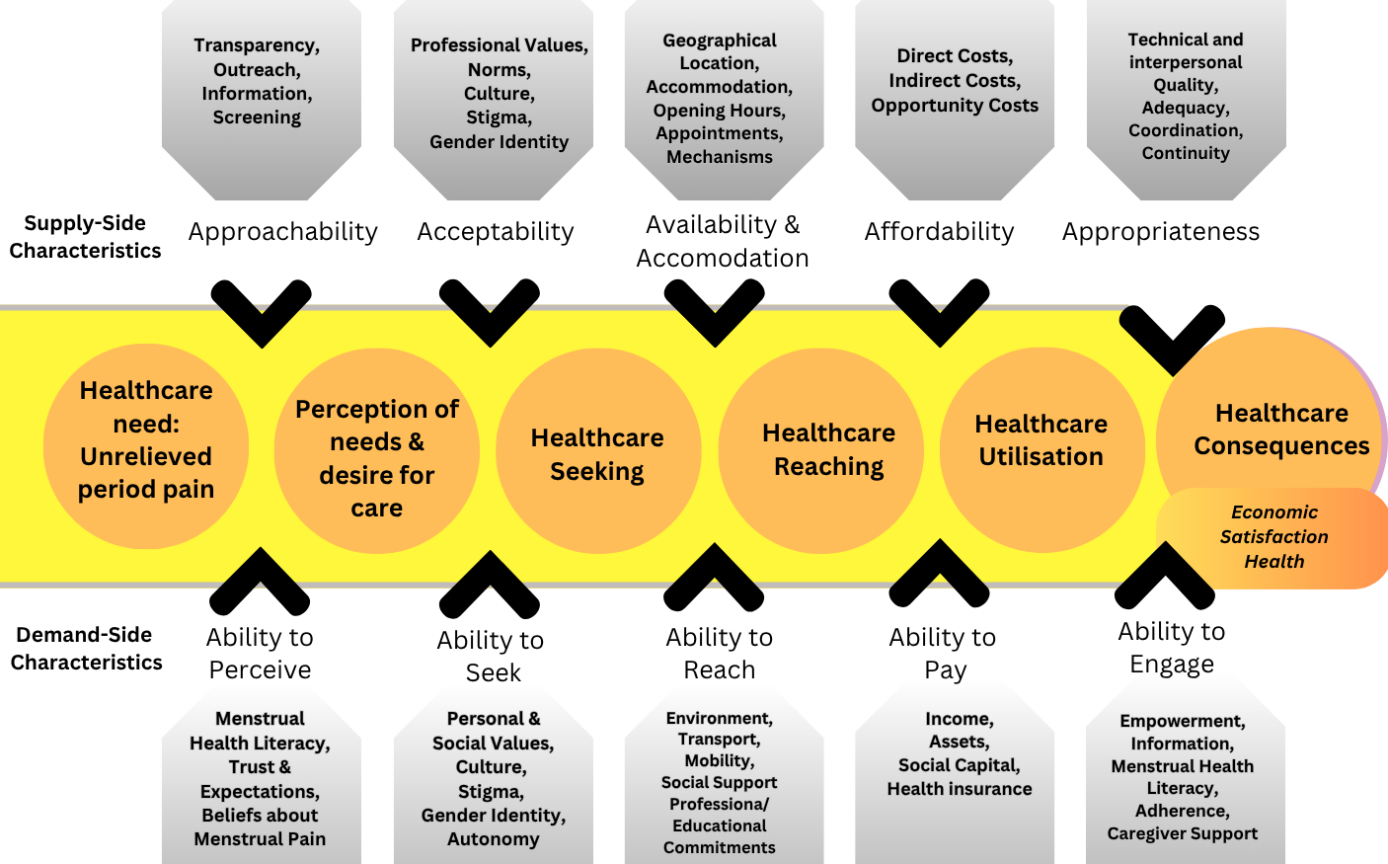
Levesque and colleagues’ (2013) Conceptual Framework of Access to Healthcare adapted for dysmenorrhoea.

Given that reasons for absences or delays in seeking healthcare are likely to have multiple causes, this framework provides a comprehensive base on which to study key factors along the pathway to accessing care and exploring these gaps in the context of dysmenorrhoea (see figure 1). These factors related to the concept of healthcare access for dysmenorrhoea are likely to be expansive and include factors from the individual level (eg, menstrual health literacy, socioeconomic status), social, cultural and environmental levels (eg, menstrual stigma, social support), as well as factors related to the healthcare system which the individual must navigate (eg, availability, outreach, affordability and perceived quality of care). The factors outlined in figure 1 are based on Levesque’s original framework and supplemented with factors identified in previous literature in dysmenorrhoea and care access.

### Rationale for scoping review

For many, unmanaged dysmenorrhoea constitutes an unmet health need that actively impacts the everyday lives of up to 91% of the 1.8 billion individuals globally who menstruate.^39^ Successfully accessing healthcare for dysmenorrhoea and fulfilling this unmet need are complex and multifactorial issues, where economic concerns, stigma, illness beliefs, low menstrual health literacy and health system restraints are all likely to interfere with the provision and uptake of care. As many HICs have recently published Women’s Health Strategies that emphasise the importance of empowering all individuals who menstruate to seek support when they struggle to manage menstrual pain, it is crucial to comprehensively assess which factors may impede healthcare access. This scoping review will seek to synthesise all relevant evidence and comprehensively map the pathway from *perceiving pain* to *fulfilling the healthcare need* to appraise how healthcare systems in HICs process dysmenorrhoea, which barriers exist to impede access and affect quality of care, and to identify key factors affecting healthcare access for dysmenorrhoea.

## Methods and analysis

### Study design

This study will constitute a scoping review, which refers to a broad form of systematic evidence synthesis that seeks to map the breadth of a particular topic.^40^ This scoping review protocol has been prepared in accordance with the Joanne Briggs Institute’s (JBI) guidance for scoping reviews^41^,^43^ based on the initial guidelines of Arksey and O’Malley.^44^ This guidance sets out a six-stage framework for conducting a scoping review, which will be used to structure the review process: (i) identifying the research question(s); (ii) identifying the relevant studies; (iii) study selection; (iv) charting the data; (v) collating, summarising and reporting results; and (vi) conducting expert consultation. The process of each stage will be outlined in the following sections.

This protocol has been developed using the Preferred Reporting Items for Systematic Reviews and Meta-Analyses Protocols (PRISMA-P) checklist^45^ and the full scoping review will be produced in line with the PRISMA-ScR extension for Scoping Reviews. Scoping reviews are often iterative processes, where changes to the original study design may be necessary to fully address the broader nature of the scoping review research question(s). The review protocol was initially preregistered on Open Science Framework as a preprint in June 2024. This protocol contains the same information as the preregistered file but contains additional information on the planned grey literature search. Any changes and deviations from this protocol will be documented in the published scoping review.

### Stage (i): identifying the research question(s)

This scoping review has been designed using the Population-Concept-Context (PCC) framework of the JBI,^43^ which provided the necessary scope to form two specific research questions (see table 1).

**Table 1:** Summary of review of PCC framework, objectives and research questions

Population	Individuals between the stages of menarche and menopause who currently or previously experience(d) dysmenorrhoea.
Concept	Literature that describes any dimension of healthcare access for the management of dysmenorrhoea as per the dimensions outlined by Levesque and colleagues.
Context	High-income countries that are members of the Organisation for Economic Co-Operation and Development (OECD); any setting, whether clinical or non-clinical.
Research aims	To identify characteristic factors that affect healthcare access for dysmenorrhea in high-income countries across both demand-side and supply-side characteristics of Levesque’s (2013) conceptual framework of access to healthcare. To explore perceptions of the quality and appropriateness of healthcare interactions for dysmenorrhoea, where care was accessed.
Research questions	What are the demand-based and system-based characteristic factors affecting healthcare access for dysmenorrhoea in HICs? How do individuals with dysmenorrhoea perceive the quality and appropriateness of healthcare interactions for the condition?

HICs, high-income countries.

A preliminary search of PubMed, PROSPERO and the Cochrane Database of Systematic Reviews was conducted in February 2024 and repeated in January 2025 to ensure no current systematic reviews or scoping reviews on the topic were underway.

### Stage (ii): identifying relevant studies

#### Types of sources

This scoping review will consider both published and grey literature, such as reports from governmental bodies and NGOs. Sources eligible for screening comprise quantitative, qualitative and mixed-methods studies, opinion, commentaries and editorial papers, as well as guidance documents, policy and/or organisation reports relevant to menstrual pain and healthcare access.

#### Inclusion criteria

The following inclusion criteria for all articles will be used to inform the searching and screening processes:

- Original studies reporting on experiences of dysmenorrhoea that explicitly discuss any facet of ‘healthcare access’ at any stage, as defined within the Conceptual Framework of Access to Healthcare by Levesque and colleagues.^38^ This includes cases along all categories of access, from not seeking healthcare for dysmenorrhoea to successful healthcare access.
- Participants within studies may range between the stages of menarche and menopause with current or previous experiences of dysmenorrhoea (whether primary or clinically diagnosed as secondary).
- Research pertaining to HICs according to membership of the OECD. A full list of OECD HICs is available in online supplemental appendix C.
- Research published in English.
- All quantitative, qualitative and mixed-methods research approaches.
- Reflective publications (eg, commentaries, editorials, opinion pieces, etc.).

No date limitations will be imposed on the searching process.

#### Exclusion criteria

- Research focusing on contraceptive use *only* without reference to dysmenorrhoea, as this does not fit the scope of the current review.
- Review articles: where systematic and/or narrative reviews emerge within the searching process, the relevant studies contained within the reviews will be screened for inclusion.

#### Search strategy

##### Peer-reviewed sources

The databases selected for searching in this review are PubMed, CINAHL, PsycINFO and Web of Science, as these databases comprehensively cover the interdisciplinary scope (health psychology, biomedical sciences) of the objectives. An initial search of PubMed was undertaken to identify articles on healthcare access for dysmenorrhoea and to aid the development of the search strategy. Keywords within titles and abstracts of relevant articles, and the index terms used to describe the articles, were used to develop a draft search strategy for PubMed (see online supplemental appendix A). A university librarian supported the initial development of the draft search strategy, which was piloted on PubMed to assess goodness of fit for search terms and potential results. The full search strategy, including all identified keywords and index terms, was adapted for each included database and/or information source and compiled in September 2024. Peer-reviewed searching across all listed databases was completed in October 2024.

To ensure comprehensive inclusion of individuals with experiences of menstrual pain, our search strategy included keywords for specific conditions, like endometriosis, adenomyosis and pelvic inflammatory disease. We note that dysmenorrhoea may be a symptom of these conditions to varying degrees and will screen all papers located through our searching for those with an explicit focus on dysmenorrhoea.

##### Grey literature searching

Searching will also include grey literature sources. Our preregistered protocol noted that grey literature would be identified through advanced Google searches of data from menstrual health organisations and governmental bodies across HICs. To supplement this approach, a Python-based web scraping technique will be used to extract PDFs directly from websites of relevant organisations. Web scraping involves locating and extracting relevant data from listed websites using software code.^46^ A list of relevant websites, including menstrual health charities, organisations, public health sites, departments of health and corporate bodies operating under the theme of menstrual health and well-being, will be collated. This list will comprise current partners of Menstrual Hygiene Day and other menstrual health initiatives, as recommended by Barrington *et al*.^47^ Organisations will also be identified through soliciting input from social media and through emails to academics working in each of the HICs under study and who have recently published on a related topic. A call will also be placed in stakeholder networks pertaining to research in menstrual health for evidence, such as the Menarche, Menstruation, Menopause, Mental Health (4M) Consortium.^48^ Finally, other organisations and potential bodies of grey literature will be identified using Google searches.

Once a comprehensive list of websites is compiled, our web scraper will recursively crawl through each of the websites one by one. It will then download all PDF files on the website, up to a recursion depth of 2, meaning that where websites contain links to other websites, it will follow links within the same domain up to two levels of depth.

For each downloaded PDF, the scraper then checks whether the document contains at least one keyword from each of two categories: (1) menstrual pain keywords (eg, ‘period pain’, ‘dysmenorrhea’) and (2) healthcare keywords (eg, ‘doctor’, ‘healthcare’, ‘GP’). If a PDF does not contain at least one keyword from both categories, it is deleted. If it does, it is saved and passed to the screening stage.

### Stage (iii): study selection

One researcher (FNC) will conduct searches of each database. Following the initial search, all citations will be collated and uploaded into Refworks^49^ and duplicates will be removed. Citations will then be uploaded to Covidence^50^ for screening by title and abstract. At least 20% of all titles and abstracts of search results will be doubly screened by two members of the research team (FNC, SB). Cases of ambiguity will be included or excluded based on the decision of a third reviewer from the research team. The full text of all potentially relevant sources will be retrieved and screened, with a minimum of 20% of full texts to be doubly screened by two members of the research team (FNC, SB) based on the inclusion criteria. Reasons for exclusion of full texts will be recorded and reported in the full scoping review. Any disagreements that arise between the reviewers at each stage of the process will be resolved through discussion with a third reviewer from the research team. The results of the search and the study inclusion process will be reported in full in the final scoping review and presented in a Preferred Reporting Items for Systematic Reviews and Meta-analyses extension for scoping review (PRISMA-ScR) flow diagram.^51^

### Stage (iv): extracting the data

A draft data extraction form was developed by the research team to target characteristics relevant to the two research questions (see online supplemental appendix B). This tool will be piloted on three studies initially and will be refined prior to use for the screened results. Any changes to the draft data extraction form available here will be reported in the published scoping review. Data will be extracted from papers included in the scoping review by the first author, with 20% of papers being extracted by two members of the research team (FNC, SB) separately using the data extraction form to examine consistency. Data extraction will comprise specific details about the participants, components of Levesque’s conceptual framework included in the source (see figure 1), the study/source context, the methods and key findings and results relevant to the review questions. If appropriate and where necessary, additional or missing data will be requested from authors of included papers. The data extraction form will be housed in the University of Stirling’s Microsoft Teams Online workspace to enable collaborative extraction.

### Stage (v): collating, summarising and reporting the results

Results will describe the screening and selection process with the visual aid of a PRISMA flow diagram. Quantitative details will be summarised, such as data relating to the prevalence and use of healthcare for dysmenorrhoea, using frequency counts and descriptive statistics. Qualitative data will be summarised using content analysis in NVivo^52^ to create overall categories of data related to the concepts in Levesque’s framework. A narrative summary will then accompany the charted results in relation to the specific review questions. An overview of the care pathway for dysmenorrhoea will then be presented, as per McCollum and colleagues.^53^

### Stage (vi): expert consultation

This protocol will be presented at the inaugural 4M Conference 2024 to generate insights from both academic and non-academic stakeholders and experts in the field of menstrual health. Other experts will be contacted over email during 2024–2025 and asked to share any data or information they may have to contribute to the search for relevant data or organisations for grey literature. It is anticipated that results of the review will also be shared with key experts through the 4M Consortium network and related stakeholder networks for expert consultation at key milestones in the review process.

### Patient and public involvement

This review does not feature patient and public involvement.

## Ethics and dissemination

This scoping review does not require ethical approval, as all data will be collected from available sources. Results from this study will be disseminated using peer-reviewed publications, conference presentations and knowledge transfer and exchange opportunities with stakeholder networks.

## Supplementary material

10.1136/bmjopen-2025-100273 online supplemental file 1
