# Supplementary material for: Factors affecting healthcare access for dysmenorrhoea: a scoping review protocol
Source: BMJ Open. 2025 Aug 6;15(8):e100273. doi: 10.1136/bmjopen-2025-100273 (PMC12336568; doi:10.1136/bmjopen-2025-100273)
Supplement: online supplemental file 1 [file bmjopen-15-8-s001.docx]

# Appendices

### Appendix A: Initial search strategy for PubMed

Population

MeSH terms

("dysmenorrhea"[MeSH Terms] OR "endometriosis"[MeSH Terms] OR “adenomyosis [MeSH Terms] OR “polycystic ovar* syndrom” [MeSH Terms] OR “uterine fibroid* [MeSH Terms] OR “pelvic inflammatory disease”[MeSH Terms]

Keywords

("Period Pain"[Title/Abstract] OR "Menstrual pain"[Title/Abstract] OR "Painful menstruation"[Title/Abstract] OR "painful period*"[Title/Abstract]))

NOT (“Sexually transmitted disease*” OR “*fertility” OR “Hirsut*” OR “Urinary” OR “Cancer”)

AND

Concept

((("Healthcare Access"[Title/Abstract] OR "Care Access"[Title/Abstract] OR "Medical Care"[Title/Abstract] OR "Healthcare Utilisation"[Title/Abstract] OR "Healthcare Services"[Title/Abstract] OR "Clinician"[Title/Abstract] OR "Healthcare Professional"[Title/Abstract] OR "Practitioner"[Title/Abstract] OR "Health Concerns"[Title/Abstract] OR "Health-Seeking"[Title/Abstract]) OR ("general practitioner*"[MeSH Terms] OR ("health personnel"[MeSH Terms] OR "diagnosis"[MeSH Terms]) OR "Patient Acceptance of Healthcare"[MeSH Terms]) OR "Delivery of healthcare"[MeSH Terms] OR "Healthcare Costs"[MeSH Terms] OR "Health Services Accessibility"[MeSH Terms] OR "health knowledge attitudes practice"[MeSH Terms] OR "Attitude to Health"[MeSH Terms] OR "health belief*"[MeSH Terms] OR "culture"[MeSH Terms] OR "Social Capital"[MeSH Terms] OR "Health Insurance"[MeSH Terms] OR "Health Service Needs and Demands"[MeSH Terms] OR "Help-Seeking Behaviour"[MeSH Terms] OR "Help-Seeking Behavior"[MeSH Terms]))

Context

**Titles & Abstracts will be screened to ensure studies emanate from HICs identified in Appendix C.*

### Appendix B: Data extraction instrument

| Study Details | | | | | | | | | |
| --- | --- | --- | --- | --- | --- | --- | --- | --- | --- |
| **Data Item** | **Study ID** | **Extractor** | **Date of Extraction** | **Title** | **Author(s)** | **Eligible for Review?** | **Reason for Exclusion** | **Article Type** | **Year of Publication** |
| Coding Guidance | [number format only] | [Initials] | [DD/MM/YYYY] | [As published] | [Up to 3; Surnames][  Beyond 3; Surnames et al] | [Yes;No] |  | [Unpublished, peer-reviewed original research, editorial, commentary, non-peer reviewed report] | [yyyy] |

| Study Details | | | | | | | |
| --- | --- | --- | --- | --- | --- | --- | --- |
| **Source/ Journal** | **Country (Specific Setting)** | **Specific Clinical Setting?** | **Which Research Question is this relevant for?** | **Study Design** | **Study Objective** | **Population** | **Use of Primary/Secondary Data** |
| [As published; website] |  | [Insert if given; GPs/Womens' Health Clinics/Hospital etc] | [RQ1, RQ2, Both] | Quantitative; Qualitative; Mixed-Methods; Case Study; Commentary; Editorial; Report of previously unpublished data [e.g Charity report] |  |  |  |

| Participants | | | | |
| --- | --- | --- | --- | --- |
| **Sample Size** | **Age** | **Primary/Secondary Dysmenorrhea?** | **If Secondary, What diagnoses?** | **Sociodemographic Information** |
|  | (Indicate using [] what metric used e.g average, mode range) |  | [Otherwise NA] | [Typically available from tables if in doubt. Gender identity, nationality, race/ethnicity, professions, educational level, SES classification, urban/rural resident] |

| Methods | | | Results | | |
| --- | --- | --- | --- | --- | --- |
| **Methods Used** | **Outcome measures (If quantitative)** | **Scales (if used)** | **Results of the study** | **Number of Participants who successfully accessed healthcare if available; % of total** | **Reasons for seeking healthcare (if given)** |
| [Data collection methods; analysis] |  | [Upper-Lower limits. Indicate using [] whether high or low scores are considered 'good'] | [Insert brief summary of key findings for research question] |  |  |

| Supply-Side Characteristics | | | | |
| --- | --- | --- | --- | --- |
| **Approachability** | **Acceptability** | **Availability & Accomodation** | **Affordability** | **Appropriateness** |
| *[Insert details/evidence related to this dimension as it served as either a barrier or an enabler of access in the context; Otherwise NA]* | *[Insert details/evidence related to this dimension as it served as either a barrier or an enabler of access in the context; Otherwise NA]* | *[Insert details/evidence related to this dimension as it served as either a barrier or an enabler of access in the context; Otherwise NA]* | *[Insert details/evidence related to this dimension as it served as either a barrier or an enabler of access in the context; Otherwise NA]* | *[Insert details/evidence related to this dimension as it served as either a barrier or an enabler of access in the context; Otherwise NA]* |

| Demand-Side Characteristics | | | | |
| --- | --- | --- | --- | --- |
| **Ability to perceive** | **Ability to perceive** | **Ability to perceive** | **Ability to perceive** | **Ability to perceive** |
| *[Insert details/evidence related to this dimension as it served as either a barrier or an enabler of access in the context; Otherwise NA]* | *[Insert details/evidence related to this dimension as it served as either a barrier or an enabler of access in the context; Otherwise NA]* | *[Insert details/evidence related to this dimension as it served as either a barrier or an enabler of access in the context; Otherwise NA]* | *[Insert details/evidence related to this dimension as it served as either a barrier or an enabler of access in the context; Otherwise NA]* | *[Insert details/evidence related to this dimension as it served as either a barrier or an enabler of access in the context; Otherwise NA]* |

| Access Characteristics | Healthcare Consequences | | | | | Conclusions |
| --- | --- | --- | --- | --- | --- | --- |
| **Other details for healthcare access not otherwise categorised** | **Treatment approach (if given)** | **Reason for treatment approach (if given)** | **Perception of treatment approach** | **Characteristics of the experience accessing healthcare for dysmenorrhea** | **Information given to participants during the healthcare consultation** | **Key conclusions of the authors** |
| *[Insert details/evidence related to this dimension as it served as either a barrier or an enabler of access in the context; Otherwise NA]* | *[Insert details/evidence related to this dimension as it served as either a barrier or an enabler of access in the context; Otherwise NA]* | *[Insert details/evidence related to this dimension as it served as either a barrier or an enabler of access in the context; Otherwise NA]* | *[Insert details/evidence related to this dimension as it served as either a barrier or an enabler of access in the context; Otherwise NA]* | *[Insert details/evidence related to this dimension as it served as either a barrier or an enabler of access in the context; Otherwise NA]* | *[Insert details/evidence related to this dimension as it served as either a barrier or an enabler of access in the context; Otherwise NA]* | *[Insert details/evidence related to this dimension as it served as either a barrier or an enabler of access in the context; Otherwise NA]* |

### Appendix C: Full List of HICs per OECD Membership

Australia Slovakia

Austria Slovenia

Belgium Spain

Canada Sweden

Chile Switzerland

Colombia Turkey

Costa Rica United Kingdom

Czechia United States of America

Denmark

Estonia

Finland

France

Germany

Greece

Hungary

Iceland

Ireland

Israel

Italy

Japan

Republic of Korea

Latvia

Lithuania

Luxembourg

Mexico

Netherlands

New Zealand

Norway

Poland

Portugal
