# Supplementary material for: Factors affecting healthcare access for dysmenorrhoea: a scoping review protocol
Source: BMJ Open. 2025 Aug 6;15(8):e100273. doi: 10.1136/bmjopen-2025-100273 (PMC12336568; doi:10.1136/bmjopen-2025-100273)
Supplement: Supplementary file 2 [file bmjopen-2025-100273.reviewer_comments.pdf]

PEER REVIEW HISTORY

BMJ Open publishes all reviews undertaken for accepted manuscripts. Reviewers are asked to complete a checklist review form and are provided with free text boxes to elaborate on their assessment. These free text comments are reproduced below.

ARTICLE DETAILS

Title (Provisional)

Factors Affecting Healthcare Access for Dysmenorrhea: A Scoping Review Protocol

Authors

Ní Chéileachair, Fódhla; Caes, Line; Belfield, Sophie; Bartl, Marion; Durand, Hannah

---

VERSION 1 - REVIEW

**Reviewer**	**1**
**Name**	**Hellman, Kevin**
**Affiliation**	**NorthShore University HealthSystem**
**Date**	**29-Apr-2025**
**COI**	**None**

This manuscript is a protocol for a scoping review on access to healthcare (???) for women with dysmenorrhea. However, I insert question marks in the first sentence here because the term “characteristics” for healthcare access seems vague to the reviewer. The second concept being reviewed is “How have cases of dysmenorrhea been approached and managed in clinical settings in HICs?” This is more concrete, but it is likely not entirely reflected in the current literature and requires extensive investigative work. Menstrual pain and the lack of effective management are significant issues—thus, an article on this topic is important, but will likely become incredibly expansive without leading to clear solutions. Therefore, the authors might benefit from more focus. Key strengths include the planned use of guidelines for scoping reviews, registration, consistency checking, and librarian assistance. Thus, addressing the issues above and the issues documented below would be prudent to increase the planned value of their manuscript.

Major issues:

Menstrual pain is a gynecological problem, but they have no gynecologists as part of their research team and lack involvement from patient advocacy groups.

It is unclear how they will conduct web scraping in their protocol.

The rationale for including pelvic inflammatory disease is unclear, as it is not a significant cause of dysmenorrhea.

Just because healthcare does something does not mean it's a good idea (it might be good or bad depending on the prior research).

I am unaware whether 4M conference provides much content on dysmenorrhea or hosting content experts. I didn't find anything suggesting they discussed menstrual pain or had content experts on menstrual pain at this conference

The management and treatment of primary dysmenorrhea is quite distinct from secondary dysmenorrhea—the authors should probably specialize.

VERSION 1 - AUTHOR RESPONSE

Reviewer's Response and Comments

We would like to sincerely thank Reviewer 1 for their thoughtful comments and for allowing us to improve the quality of the manuscript and its research. Below we include a point-by-point address of each comment and highlight where relevant changes have been made to the manuscript. We are grateful to Reviewer 1 for taking the time to review our protocol.

This manuscript is a protocol for a scoping review on access to healthcare (???) for women with dysmenorrhea. However, I insert question marks in the first sentence here because the term "characteristics" for healthcare access seems vague to the reviewer.

> Response. We agree that the initial terminology used is vague and clarity is needed. Our scoping review seeks to clarify all factors available in the literature along the pathway to accessing healthcare, as defined by Levesque and colleagues' framework. This work is being undertaken to highlight why some may seek help, why some may not and where some individuals who feel they might benefit from healthcare might fall off the pathway towards accessing it. We believe this study will be expansive but that key factors can be effectively categorised along the framework to map impediments in the pathway to care in a logical and comprehensive way. We have now amended our title to 'Factors affecting healthcare access for dysmenorrhea" to ensure greater clarity of subject for our readers, as well as using the term 'factors' throughout the text.

The second concept being reviewed is "How have cases of dysmenorrhea been approached and managed in clinical settings in HICs?" This is more concrete, but it is likely not entirely reflected in the current literature and requires extensive investigative work. Menstrual pain and the lack of effective management are significant issues—thus, an article on this topic is important, but will likely become incredibly expansive without leading to clear solutions.

Response. We believe clarifying this point will improve the manuscript. This question was designed to flesh out the final element of Levesque's framework, where studies and reports accounting for the appropriateness of healthcare encounters and outcomes from the perspective of the patient can be collated and commonalities can be summarised. Answering this research question will also highlight important aspects of care from the perspective of the patient, like shared-decision making, accounting for patients' preferences, provision of clear information and opportunities to ask questions. We have updated the description of this research aim and question, which has now been restated to the following:

"To explore the appropriateness and quality of interactions with healthcare provides from the perspective of those who accessed care for dysmenorrhea"

RQ2: How do individuals with dysmenorrhea perceive the quality and appropriateness of healthcare interactions for the condition?

We agree with Reviewer 1 that more investigative work in this area is needed. We are hopeful that our review will pinpoint specific gaps that remain to be more thoroughly researched within this framework.

Therefore, the authors might benefit from more focus. Key strengths include the planned use of guidelines for scoping reviews, registration, consistency checking, and librarian assistance. Thus, addressing the issues above and the issues documented below would be prudent to increase the planned value of their manuscript.

Response. We appreciate the encouragement of Reviewer 1 in identifying these research strengths. By reframing our title and aims throughout the manuscript, we hope that the focus of our review is now clear.

Major issues:
Menstrual pain is a gynecological problem, but they have no gynecologists as part of their research team and lack involvement from patient advocacy groups.

Response. It is true our research team does not have a gynaecologist and would benefit from this perspective. We are approaching this research topic from the discipline of health psychology, focusing on experiences, behaviours, motivations, and capacities in navigating specific health needs. We are fortunate to have a research team with specific expertise informing this review, including expertise in pediatric pain experiences and self-management behaviours for chronic illnesses, particularly related to women's health concerns. Our team also includes two PhD students with a shared focus on menstrual health and a computer scientist to guide our approach to webscraping for grey literature. This work represents the first stage of a larger project into healthcare needs for

dysmenorrhea. We are building connections with patient advocacy groups in the UK and Ireland and will seek their input to overall project as it progresses.

It is unclear how they will conduct web scraping in their protocol.

Response. We have now provided greater detail on the approach in designing the webscraper and the steps taken to search through identified websites (See below). The code was written by a member of the research team. The scraper downloads all PDFs on the website, checks for relevance using a modified keyword search, and deletes irrelevant PDFs. All code will also be made available once the finalised review is published, with all identified PDFs available for view and the breakdown of those included/excluded.

"Once a comprehensive list of websites is compiled, our webscraper will recursively crawl through each of the websites one-by-one. It will then download all PDF files on the website, up to a recursion depth of 2, meaning that where websites contain links to other websites, it will follow links within the same domain up to two levels of depth. "

For each downloaded PDF, the scraper then checks whether the document contains at least one keyword from each of two categories: (1) menstrual pain keywords (e.g., "period pain", "dysmenorrhea") and healthcare keywords (e.g., "doctor", "healthcare", "GP"). If a PDF does not contain at least one keyword from both categories, it is deleted. If it does, it is saved and passes to the screening stage. "

The rationale for including pelvic inflammatory disease is unclear, as it is not a significant cause of dysmenorrhea.

Response: Thank you for this comment. Following a number of discussions within the research team, we opted to ensure the searches were as inclusive as possible of conditions that may incur secondary dysmenorrhea. Similar search terms have previously informed similar reviews, such as Fryer, Mason-Jones & Woodward's (2024) scoping review study 'Understanding diagnostic delay for endometriosis: A scoping review using the social-ecological framework". We agree that not all individuals with a diagnosis of pelvic inflammatory disease will have dysmenorrhea as part of the condition, however it is included here to ensure we were not intentionally overrepresenting other conditions, such as endometriosis, on which several papers have been published. All papers related to conditions like pelvic inflammatory disease that do not explicitly refer to menstrual pain will be screened out during the process.

Just because healthcare does something does not mean it's a good idea (it might be good or bad depending on prior research).

Response: We agree that this is an important point to clarify. We have adapted the introduction and the rationale of the review to explicitly note that not all individuals with dysmenorrhea will require medical attention or intervention (Page 7, Lines 116 onward)

Commented [LC1]: add the page numbers, likely too much to add the changed sentences , so here ok to just refer to the pages changed

I am unaware whether 4M conference provides much content on dysmenorrhea or hosting content experts. I didn't find anything suggesting they discussed menstrual pain or had content experts on menstrual pain at this conference

Response: 4M is a relatively new research network but does have members who have expertise in menstrual pain and dedicated half of the first day of conference 2024 to the management of menstrual bleeding and pain experiences. The conference programme is available to view here: https://4mhealth.uk/activities/4m-conference-2024/provisional-programme/. We are also fortunate to have access to the 4M stakeholder group through this network, including patient advocacy groups. In gathering grey literature sources, we have also contacted researchers and experts in dysmenorrhea and related fields from all OECD countries.

The management and treatment of primary dysmenorrhea is quite distinct from secondary dysmenorrhea—the authors should probably specialize.

Response: We appreciate the distinction raised between primary and secondary dysmenorrhea, particularly regarding differences in treatment and management. However, it is important to note that much of the existing literature cannot definitively describe participants as having primary dysmenorrhea, given the nature of their study designs and the absence of diagnostic confirmation. This reflects a broader issue in the field: the considerable barriers to diagnosis for conditions underlying secondary dysmenorrhea (e.g., endometriosis, adenomyosis), which often result in individuals remaining undiagnosed for years. As such, many participants in the included studies may be describing symptoms that are, in fact, secondary in origin. We therefore believe that restricting the review to studies explicitly focusing on primary dysmenorrhea would risk excluding a substantial proportion of relevant experiences and limit the applicability of our findings to real-world healthcare interactions, where diagnostic uncertainty is common. For this reason, we maintain a broader

inclusion approach while clearly acknowledging the limitations around diagnostic specificity in the included studies.
